# Music preference and political orientation have similar (but independent) effects on social attraction

**DOI:** 10.3389/fcogn.2026.1838689

**Published:** 2026-09-17

**Authors:** Olivia Jewell, Lauren Stewart, Keon West, Jacques Launay

**Affiliations:** Department of Psychology, Goldsmiths University of London, London, United Kingdom

**Keywords:** attraction, music, music preferences, politics, social attraction

## Abstract

**Introduction:**

Similarities between people, including similarities in music preference, can increase social attraction. It has been suggested that the relationship between music preference similarity and social attraction operates indirectly via attributes which music preference is signaling. In other words, individuals may infer other shared traits or values from music preference, which may reflect broader similarity. The current study examined whether music preference similarity and political orientation similarity interact to predict social attraction or whether music preference similarity has an independent effect on social attraction.

**Method:**

In an online, between-groups study participants were assigned to groups in which musical and political similarity with a target person were manipulated. Participants then reported their social attraction towards the target. If music preference similarity influences social attraction primarily by signaling political orientation, an interaction between musical similarity and political similarity on social attraction would be expected, with no independent effect for music preference alone.

**Results:**

The current study found independent positive effects of music preference similarity and political orientation similarity on social attraction, with no interaction between the two independent variables.

**Discussion:**

Findings indicate that music preference similarity and political orientation similarity are influencing social attraction separately, suggesting that shared music taste is not just acting as an indicator of other similarities. This research has implications for the use of music preferences to bridge differences between individuals.

## 1 Introduction

Similarity-attraction research has demonstrated that similarities between individuals increase social attraction (Byrne et al., 1966; Condon and Crano, 1988; McCroskey et al., 2006; Singh, 1973). For this study, social attraction is based on McCroskey and colleagues' definition of social attraction as one aspect of interpersonal attraction, encompassing general positive feelings toward a person, liking, and feelings of friendship (McCroskey and McCain, 1974). Early research by Byrne (1961) established that participants feel more positive toward a stranger that they shared attitudes with compared to a stranger with whom they shared no attitudes. Byrne and Nelson (1965a,b) demonstrated that the number of *attitudes people share is positively related to the degree of social attraction. This appears to* be the case regardless of how important participants rated those attitudes (Byrne and Nelson, 1965a,b). Perceptions of personality, demographic, or attitude similarity can influence how much time people are interested in spending with others (Dunlop and Beauchamp, 2011; Luther et al., 2016), communications and friendships online (Aiello et al., 2012; Baym and Ledbetter, 2009), and friendship maintenance (Kandel, 1978).

The similarity-attraction phenomenon also extends to music preference. Research on music and social attraction shows that participants tend to feel more positively toward individuals who are fans of similar music styles (Baym and Ledbetter, 2009; Knobloch et al., 2000; Launay, 2015). These findings highlight the fact that shared music preferences can positively influence attraction at the individual level. One study by Bakagiannis and Tarrant (2006) also found this effect at the group level: when two groups of participants were told that members of the other group had similar music preference to theirs, participants felt more positively about the other group compared to a control group about which they were told nothing related to music preference. Similar music preferences are also associated with friendship formation in group contexts in person and online (Aiello et al., 2012; Selfhout et al., 2009). Furthermore, people tend to associate fans of their own music preference with more positive traits and fans of disliked music with more negative traits (Lonsdale and North, 2009). This raises the question: is social attraction based on similar music preferences an independent effect, or does it occur merely because music preferences are seen as indicative of other traits? Some research has argued that music can act as a kind of “social badge” to signal other traits. For example, music is used as a predictor of traits such as personality (Rentfrow et al., 2009; Rentfrow and Gosling, 2006, 2007), ethnicity (Marshall and Naumann, 2018; Shevy, 2008), and drug use (Fried, 2003). These findings illustrate that there are strong, consistent assumptions that participants make about targets based on their music preferences.

There is also research showing correlations between music preference and political orientation (Fox and Williams, 1974; North and Hargreaves, 2007; Peterson and Christenson, 1987; Rentfrow and Gosling, 2003). For example indie music, alternative, and hop-hop tend to be preferred by liberally-leaning people, while classical, country, pop, and religious music tend to be more popular for more conservative-leaning people (Fox and Williams, 1974; Middleton and Schwär, 2017; North and Hargreaves, 2007). These correlations indicate that politics is another variable which can be associated with music. For this reason, some researchers have examined whether music can reduce prejudice associated with political conflict. One study found that when participants listen to protest songs which are critical of war and the military, their empathy toward the related outgroup increased (Ziv, 2019). Political intergroup bias and prejudice were reduced when participants were exposed to music intended to unify during times of crisis (Bodner and Gilboa, 2009) or a Holocaust song (Bodner and Bergman, 2017) or pro-integration music (Greitemeyer and Schwab, 2014). Unity songs have also been found to facilitate feelings of compromise (Ziv and Shoval, 2025). Despite research indicating that music can work to reduce political tension, there is less published research examining whether shared music preference increases social attraction because it can signal political orientation, and thus membership of an in-group, or if there is an independent effect of music preference on social attraction.

Across three studies, Boer et al. (2011) illustrated that similarity in value orientation is a mediator of the relationship between music preference similarity and social attraction. The first two studies found that perceived values mediated the relationship between music preference and social attraction. However, the third study examined this finding in undergraduate roommate pairs but did not find a significant correlation between music preferences and value similarity. Although this study produced some evidence that the relationship between music preference and social attraction could be mediated by values, these mixed results and absence of a direct comparison between shared music tastes and shared values leave some ambiguity about whether music taste is acting as an indicator of other similar values (Boer et al., 2011).

Value and political orientation correlate (Caprara et al., 2006; Knutsen, 1995; Schwartz et al., 2010; Van Deth and Geurts, 1989) and both are of relevance to the current study, which explores the relationship between political orientation, music preference, and attraction. The research question of interest here is whether perceived similarity in music preferences predicts social attraction toward a target independent of perceived similarity in political orientation.

In the following study participants were assigned to one of four different conditions and presented with a fictional target named Kate. In each condition, participants' musical and political similarity with Kate were manipulated and participants were asked to rate their perceived social attraction toward Kate. This was done to establish whether high vs. low similarity in music preference and high vs. low similarity in political orientation predicted social attraction. If music preference acts primarily as a signal for values (here, political orientation) then we would expect to find an interaction between similar music preference and similar political orientation on social attraction and no independent effect of music preference on social attraction. Conversely, if these effects are independent, no interaction will be seen.

## 2 Method

### 2.1 Participants

Participants were recruited from Prolific, an online recruitment platform for research, after completing a screening survey, the details of which are below. The final sample of participants included 255 British members of the Prolific community. A power analysis indicated that 212 participants were required for this study, so we aimed to recruit approximately this number. The minimum age was 18 with a maximum of 45 (*M* = 31.6, *SD* = 7.7). Five participants identified as non-binary, 105 identified as male, and 144 identified as female. Most participants identified as White (228) with 14 identifying as Asian, four identifying as Black/Caribbean, three identified as Middle Eastern, one participant identified as Central or South American, and three participants identified as multi-racial. There were 111 participants who self-identified as conservative and 144 who self-identified as liberal. After participants completed the study, they were redirected back to Prolific where they were reimbursed £1.40 for their participation.

### 2.2 Procedure and measures

The screening survey was distributed to British members of the Prolific community who either identified as being on the left or the right of the political spectrum. Participants were shown a list of music genres and were asked to indicate the degree to which they liked that genre. Indie and country music emerged through pilot testing as two genres stereotypically aligned with the left and right of the political spectrum, respectively. Our screening sample included a substantial number of participants who either endorsed liking country music and disliking indie music or liking indie music and disliking country music. Having these two groups of participants meant that it was possible to intentionally present participants with a version of Kate who either shared their political orientation, shared their music preference, shared both of these, or shared neither. Table 1 below displays the participant group assignments based on whether each participant preference matched Kate's profile.

**Table 1:** Participant group assignments.

Group	*N*	Kate's music preference	Participant music preference	Kate's political orientation	Participant political orientation
1 (similar music, similar politics)	68	Indie	Indie	Liberal	Liberal
		Country	Country	Conservative	Conservative
2 (dissimilar music, similar politics)	64	Indie	Country	Liberal	Liberal
		Country	Indie	Conservative	Conservative
3 (similar music, dissimilar politics)	58	Indie	Indie	Liberal	Conservative
		Country	Country	Conservative	Liberal
4 (dissimilar music, dissimilar politics)	65	Indie	Country	Liberal	Conservative
		Country	Indie	Conservative	Liberal

## 3 Results

### 3.1 Social attraction

A two-way analysis of variance (ANOVA) was run to examine social attraction scores toward Kate. The independent variables were music preference (similar or dissimilar) and political orientation (similar or dissimilar). There was a significant main effect for political orientation [*F*(1,249) = 64.49, *p* < 0.001, partial η*2* = 0.21]. When participants' political orientation was similar to Kate's, social attraction scores were 0.52 points higher than when participants' political orientation was not similar to Kate's [95% CI (0.39, 0.65), *p* < 0.001]. There was also a significant main effect for music preference [*F*(1,249) = 24.63, *p* < 0.001, partial η*2* = 0.09]. When participants had similar music preferences to Kate, they liked her significantly more than when they did not have similar music preferences to Kate, by 0.32 points [95% CI (0.20, 0.45), *p* < 0.001]. There was no significant interaction between political orientation and music preference on social attraction scores [*F*(1,249) = 2.14, *p* = 0.145, partial η*2* = 0.01]. The significant main effect for similar music preference can be seen in Figure 1.

**Figure 1:**
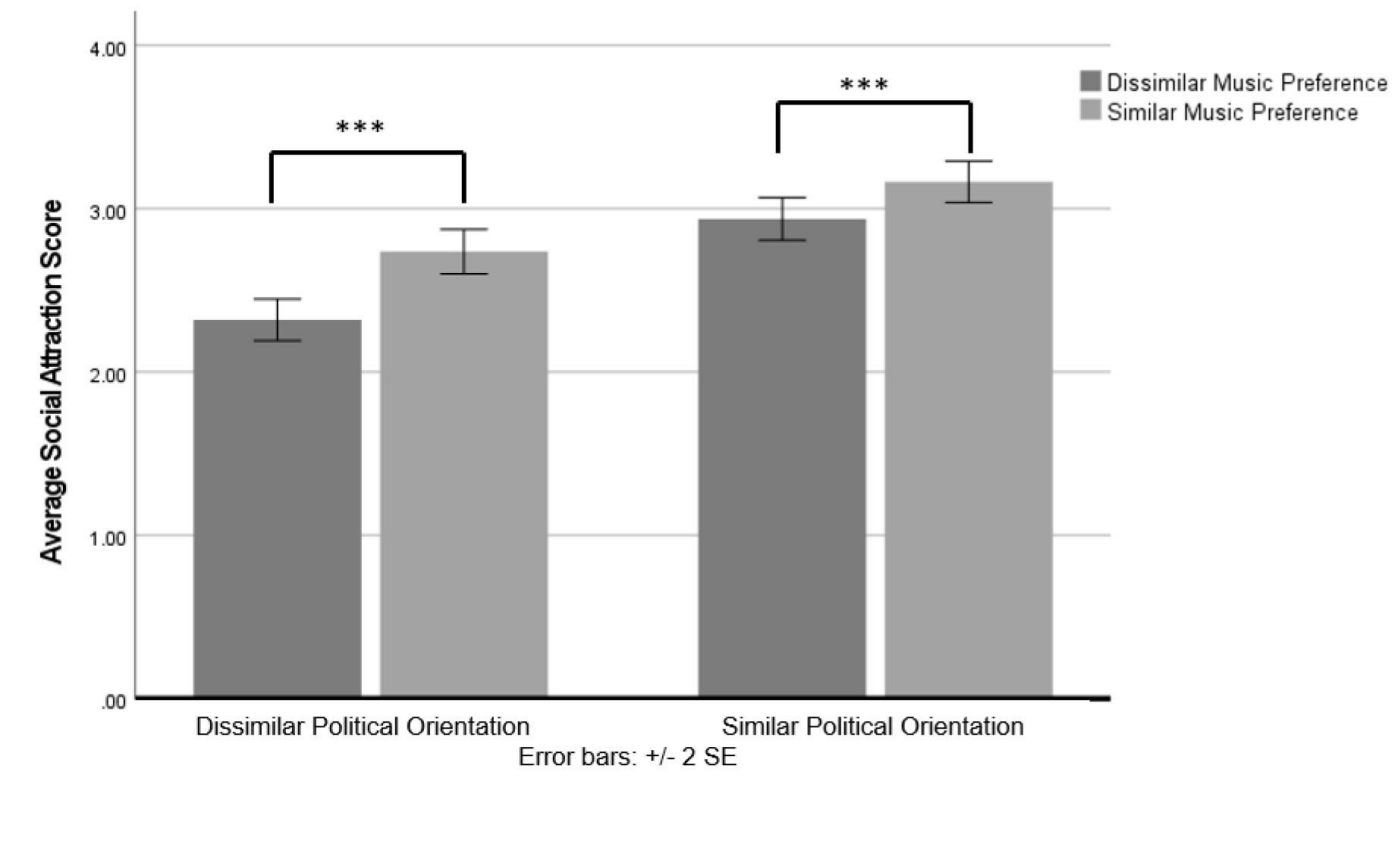
Social attraction scores by political orientation and music preference. ***significant at *p* < 0.001.

## 4 Discussion

The study above examined whether high vs. low musical similarity and high vs. low political similarity were independently influencing social attraction. The absence of an interaction between music preference similarity and political orientation similarity on social attraction, as well as the presence of main effects of both of music preference similarity and political similarity on social attraction, suggests music preference similarity has an effect on social attraction independent of political orientation similarity.

These results support previous similarity-attraction literature by illustrating that the more participants share with the target, the higher they will endorse feeling socially attracted to the target (Ajzen, 1974; Byrne and Nelson, 1965a; Montoya et al., 2008; Montoya and Horton, 2014). This is notable, as the studies by Boer et al. (2011) suggested that shared music preferences exerted effects on social attraction primarily by activity as a proxy for values (here political orientation). By collecting data on individual participants political orientation and music preferences, we were able to orthogonally vary similarity of participant and target's similarity on each of these, to empirically address the extent to which each of these can exert independent vs. interacting effects.

This finding is in contrast to the conclusions of Boer et al. (2011) who argued that value orientations were mediating the relationship between similarity in music preferences and resulting social attraction. The current study did not find an interaction between these variables which presents the possibility that values are not necessarily the mediating factor between music preference similarity and social attraction in this way. Another possibility is that value orientation and political orientation are not as closely related as can be assumed, or that music preference evokes values in a way that it does not evoke political orientation. It is also possible that values, political orientation, and music preference may have a more nuanced relationship that the current study did not capture. Future research should utilize a design which can test mediating variables more thoroughly, such as by presenting participants with more stimuli.

### 4.1 Practical implications

The independent effect of music preferences on social attraction, in light of differences in political orientation, presents implications for the use of similar music preferences for bringing people together despite salient differences (Harwood, 2017). The Contact Hypothesis suggests that social interaction between members of different groups could potentially reduce intergroup prejudice (Pettigrew and Tropp, 2006) and literature focusing on bridging differences via music tends to focus on specifically exposing participants to music of other cultures. However, there may be contexts in which this is not a straightforward process and there may be ideal or optimal conditions that need to be achieved in order for this social interaction to reduce prejudice. Those who are more highly prejudiced may also be reluctant to engage in contact with those who are different from them (Hotchin and West, 2022).

The research described here provides a rationale for music to be used as a vehicle for bringing together individuals who are different from one another without relying on exposure to novel music. Using similar music preferences to bring different people together may serve as an easier or more straightforward starting point to reducing prejudice because individuals may naturally self-select to participate in programs or activities where their favorite music is a key component. Events like music festivals where, in addition to music, there may be food, games, merchandise, and performances that highlight the culture of the music may provide an accessible context for individuals who are different to come together (Greitemeyer and Schwab, 2014; Neto et al., 2015, 2019).

While the current study presents implications for the study of music preference and the possibility for music to bring different people together, it is important to note the study's limitations. This study only used one experimental stimulus, Kate, who only represented one gender. Gender was not analyzed as a potential factor in predicting social attraction toward Kate, however, it is possible that participants were taking gender into consideration when determining how much they may have liked her. Since only one target of one gender was presented, the study was not balanced to accommodate the influence of gender in social attraction. Additionally, one of only two musical genres and one of only two political parties were included on Kate's profile, which limits the study's ability to examine the variability that exists in most people's music preferences and political opinions.

Future research could better control for variables such as gender, socioeconomic status, personality, age, or education in order to ensure that the variables influencing social attraction are only music preference and political orientation. Additionally, a future study could expand on the research above by including an additional variable which can be used to predict social attraction, such as sport's team preference or other hobbies, which may help illustrate whether the findings above are unique to music preferences, or are simply a reflection of the similarity-attraction phenomenon in general.

## 5 Conclusions

These findings expand the extant research on music preference by illustrating the independent effect of music preference on social attraction. This suggests that shared music taste might be able to influence social attraction even when it is not shown to correlate with other, indicators of shared values such as political views. This research helps provide understanding for how music preferences shape social attraction toward others and has implications for the role of music preferences in helping to bring together communities that are different from one another in other ways.

## Data Availability

The raw data supporting the conclusions of this article will be made available by the authors, without undue reservation.
